# Identification of *Bacillus megaterium* as a Probiotic and Its Action to Control Biofilms of Foodborne Pathogens

**DOI:** 10.3390/foods15152745

**Published:** 2026-08-05

**Authors:** Tatsaporn Todhanakasem, Norawachara Sanchan, Panupong Kaituam, Bo Wu

**Affiliations:** 1 School of Food Industry, King Mongkut’s Institute of Technology Ladkrabang, Bangkok 10520, Thailand; tatsaporn.to@kmitl.ac.th (T.T.);; 2 Biomass Energy Technology Research Center, Biogas Institute of Ministry of Agriculture and Rural Affairs, Renmin Rd. S 4-13, Chengdu 610041, China

**Keywords:** *Bacillus megaterium*, probiotic, biofilm, active compounds, foodborne pathogens

## Abstract

*Bacillus megaterium* isolated from coffee fermentation demonstrated potential probiotic properties against *Bacillus cereus* ATCC 11778, *Escherichia coli* ATCC 8739, and *Salmonella enterica* subsp. *enterica* serovar Typhimurium ATCC 13311 through the production of active compounds, including δ-valerolactam and ε-caprolactam. This is the first report on its potential utility in producing active compounds, forming protective biofilms, or blocking cell adhesion and aggregation of foodborne pathogenic bacteria on material mimicking the intestinal mucosa. The quorum-sensing signal molecule N-butanoyl-L-homoserine lactone, an auto-inducer for biofilm formation, was detected in the crude culture supernatant, supporting its capability for biofilm formation. Characterization assays showed *B. megaterium* to tolerate up to 6% bile salt and to survive at pH 1.5. In addition, it demonstrated negative hemolytic activity and sensitivity to antibiotics at the level. It also produced various bioactive compounds that function with therapeutic properties. Therefore, *B. megaterium* has potential to be used as a human and animal probiotic in the future.

## 1. Introduction

*Bacillus* species are well known for producing secondary metabolites or bioactive compounds with potential use in diverse applications. The genus *Bacillus* includes a considerable number of bacterial probiotics, including the commonly mentioned *B. subtilis*, *B. coagulans*, *B. pumilus*, *B. licheniformis*, and *B. clausii* [1]. They are part of the healthy microbiota of the human gut and can be applied either alone or as a microbe cocktail to prevent food spoilage and the growth of pathogenic microorganisms [2,3]. Members of this genus have also been found in Asian fermented foods based on grains, which are non-dairy fermented products [4]. *B. megaterium* is noted for rich enzyme production and strong resistance to pathogenic bacteria. It has been used as an animal probiotic for calves, lambs, shrimp, and fish to improve feed intake, daily gain, feed conversion rate, and growth performance as a whole [5,6]. It is universally accepted as a safe, non-pathogenic microorganism and holds a multi-agency recognized safety status. In addition, it produces a novel cyclic peptide compound with broad-spectrum antimicrobial activity against both Gram-positive and Gram-negative bacteria [7]. Therefore, this bacterium is of specific interest to the food industry not only for its probiotic characteristics but also because it produces antimicrobial compounds. A probiotic cocktail containing *B. megaterium*, *B. clausii*, and *B. subtilis* has proven probiotic functionality in a clinical trial [8].

Most recently, *Bacillus* species have gained attention for the potential use of probiotic biofilms as an alternative therapy for diseases caused by foodborne pathogenic bacteria. Formation of biofilms enables bacteria to resist environmental conditions, leading to successful colonization and maintenance of their population. It must be taken into consideration that colonization of the gut requires preferential adhesion of bacteria to intestinal mucosa, prolonging and stabilizing their residence, helping to exclude pathogenic bacteria by competitive inhibition or steric impediment, and possibly triggering the immune response of the host [9,10]. Spore-forming probiotics, for instance *B. megaterium*, have also emerged as a potential avenue for combating foodborne pathogenic biofilms. Co-culture of *B. megaterium* with *Listeria monocytogenes* has been demonstrated to suppress *Listeria monocytogenes* growth and expression of virulence genes of *L. monocytogenes* [11]. In addition, a *Bacillus* sp. probiotic biofilm has been reported to introduce biochemical effects such as antimicrobial and enzymatic activity, thus contributing to protection from the gastrointestinal tract and foodborne pathogenic infections [1].

The present study aims to determine the potential utility of *B. megaterium* isolated from coffee fermentation as a probiotic. The physiological assays will be conducted to reveal the capability of the isolated strain in preventing the growth and the biofilm formation of foodborne pathogenic microbes, including *B. cereus*, *Escherichia coli* and *Salmonella enterica* subsp. *enterica* serovar Typhimurium. We will also gain insight into the isolate’s production of bioactive compounds, biofilm, and cell aggregation blockages. The probiotic characteristics, including bile salt tolerance, acidic pH tolerance, hemolysis activity, and antibiotic susceptibility, will also be determined.

## 2. Materials and Methods

### 2.1. Bacterial Identification

The culture was isolated from Arabica coffee (*Coffea arabica* L.) fermented by the wet process at an organic coffee farm in Tongping, Chiang Mai, Thailand. Trifluoroacetic acid (TFA) 2.5% was dissolved in 250 µL of anhydrous acetonitrile. The bacteria were identified based on a single isolated colony. The culture was smeared on MALDI and monitored with a MALDI biotyper by Medical and Food Lab Co. Ltd., Bangkok, Thailand.

### 2.2. Bacterial Strains and Culture Conditions

The isolated *B. megaterium* strain was cultivated at 37 °C using nutrient broth (NB) or nutrient agar (NA). Additional bacterial strains were sourced from the American Type Culture Collection (ATCC). Under identical temperature conditions (37 °C), *Escherichia coli* ATCC 8739 and *B. cereus* ATCC 11778 were also propagated in NA or NB. Meanwhile, *S. enterica* subsp. *enterica* serovar Typhimurium ATCC 1331 was incubated in tryptic soy broth (TSB) or tryptic soy agar. Before initiating any experiments, all cultures were incubated overnight until reaching an optical density (OD_600_) of approximately 1.0. For long-term preservation, stocks of each strain were stored at −80 °C in their respective growth media supplemented with 20% (*v*/*v*) glycerol.

### 2.3. Antimicrobial Activity of B. megaterium

To assess the antimicrobial activity of the neutralized cell-free supernatant from *B. megaterium* culture (OD_600_ approximately 1.0), disk diffusion assays were conducted against three indicator strains: *B. cereus*, *E. coli*, and *S. enterica* subsp. *enterica* serovar Typhimurium. In short, 20 µL aliquots of the supernatant were applied to disks on inoculated agar plates, which were then kept at 37 °C for a 24-h incubation period before measuring the resulting inhibition zones. Kanamycin and erythromycin at concentrations of 100 µg/mL served as positive controls, whereas sterile distilled water was employed as the negative control. Every assay was executed in triplicate, with the gathered data subjected to statistical validation via ANOVA coupled with Duncan’s multiple range test.

### 2.4. Biochemical Compound Analysis Using Ultra-High-Performance Liquid Chromatography (UHPLC) and Quadrupole Time-of-Flight Mass Spectrometry

To characterize the biochemical constituents within the crude *B. megaterium* supernatant, an ultra-high-performance liquid chromatography (UHPLC) system (Ultimate 3000, Thermo Scientific, Germering, Germany ) coupled to a compact quadrupole time-of-flight (QTOF) mass spectrometer (Bruker, Bremen, Germany) was utilized. Separation was performed on a Bruker Intensity Solo 2 C18 column (100 × 2.1 mm). Sample preparation involved combining 0.5 mL of the culture slurry with 5 mL of LC-grade water in a 20 mL vial. Following a 5 min sonication step, extraction was executed using an agitator set to 70 °C and 700 rpm for 6 min. The filtrates were passed through 0.22 µm nylon membranes and moved to 1.5 mL vials for analysis. The UHPLC parameters included a 5 µL injection volume, a 35 °C column temperature, a flow rate of 0.40 mL/min, and a multi-step gradient profile lasting 0–26 min. Mobile phase A consisted of water with 2.5 mM ammonium formate buffer, while mobile phase B comprised methanol with 5 mM and 2.5 mM ammonium formate buffer.

Mass spectra were acquired in the range of 50–1000 *m*/*z* using data-dependent acquisition (AutoMSMS mode) in positive ion mode with electrospray ionization at 2.5 kV. Daily QTOF calibration was performed using a sodium formate standard solution. The MS data were imported into MetaboScape Software, MetaboScape 2024 (Bruker Daltonics, Bremen, Germany ) and processed with the T Rex 3D algorithm. Parameters for feature extraction included a primary ion of [M-H]^−^, an intensity threshold of 1000 counts, retention time, and mass-to-charge ratio (*m*/*z*), resulting in a comprehensive feature table. Annotated features were identified using the BrukerLocal Library 3.0 [12].

### 2.5. Biofilm Forming Ability of B. megaterium Compared with Pathogenic Strains

The quantitative approaches were employed to evaluate the capacity of selected isolates to establish biofilms. The quantitative assessment relied on a crystal violet staining method (0.1% *v*/*v* dye concentration) to measure the bacterial adhesion to polystyrene (PS) surfaces via optical density measurements at 600 nm. To initiate biofilm growth, overnight cultures of *B. megaterium*, *B. cereus*, and *E. coli* were prepared in nutrient broth (NB), while *S. enterica* subsp. *enterica* serovar Typhimurium was similarly incubated in tryptic soy broth (TSB). The following day, suspensions adjusted to an OD_600_ of approximately 1.0 were introduced into 2 mL of a 1:5 diluted solution of their respective growth media (either NB or TSB). The biofilms were subsequently cultivated at 37 °C under static conditions for three consecutive days, during which the incubation media was refreshed every 24 h. Daily monitoring of bacterial attachment involved treating the wells with a 0.1% *w*/*v* crystal violet solution for 20 min, followed by a destaining step using 95% ethanol. A spectrophotometer was then used to record the absorbance of the eluted dye at 600 nm. Individual strain capabilities were evaluated and compared based on the colorimetric intensity of the destained crystal violet [13]. All trials were executed in triplicate, and statistical significance was verified using ANOVA accompanied by Duncan’s multiple range test, applying a threshold of *p* < 0.05 to determine the validity of the null hypothesis.

### 2.6. Antimicrobial Activity of Crude Supernatant from B. megaterium Against Biofilms of Pathogenic Strains

To isolate cell pellets and clarify supernatants, overnight cultures of *B. megaterium* were subjected to centrifugation. A 0.2 mL volume of this crude supernatant was subsequently inoculated into 2 mL of overnight target cultures (*B. cereus*, *E. coli*, or *S. enterica* subsp. *enterica* serovar Typhimurium) (OD approximately 1.0, containing 10^7^–10^8^ CFU/mL). The resulting co-cultures were then incubated at 37 °C for a duration of 0 to 3 days. Biofilm development was subsequently quantified via crystal violet staining along the incubation period. Absorbance readings were gathered at 600 nm utilizing a spectrophotometer, with biofilm-forming capacity evaluated based on stain intensity. Triplicate assays were performed, and data significance was evaluated via ANOVA combined with Duncan’s multiple range test at a 95% confidence interval (*p* < 0.05).

### 2.7. Auto-Aggregation and Co-Aggregation of B. megaterium

The auto-aggregation and co-aggregation capacities of *B. megaterium* were determined following the methodology outlined by Collado et al. [14]. Biomass from an overnight culture was collected via centrifugation for 2 min. The resulting cell pellets were rinsed twice using a phosphate buffer system (pH 7.0) before being resuspended in the identical buffer to achieve a target cell density of roughly 10^7^–10^8^ CFU/mL. After documenting the initial absorbance (OD_600_) of the homogenized bacterial suspension, the samples were left undisturbed at 37 °C for a 24-h incubation period, after which the final optical density was measured. The aggregation percentage was calculated using the equation as [1 − (A_time_/A_0_) × 100], where A_time_ represents the absorbance of the mixture at 24 h and A_0_ the absorbance at time 0.

To evaluate the co-aggregation profiles of pathogenic microorganisms with *B. megaterium*, the optical density (OD) of mixed suspensions was measured following an overnight co-incubation with *B. cereus*, *E. coli*, or *S. enterica* subsp. *enterica* serovar Typhimurium. Briefly, *B. megaterium* bacterial suspensions were generated according to the previously detailed protocol and combined with an equal volume (500 µL) of the respective target pathogenic microbe. The resulting mixtures were then incubated at 37 °C for 24 h, after which percent co-aggregation was evaluated as (A_pathogen_ + A*_B. megaterium_*)/2 − (A_mix_)/(A_pathogen_ + A*_B. megaterium_*)/2) × 100, where A_pathogen_ and A*_B. megaterium_* represent the absorbances of the respective pure cultures and A_mix_ the absorbance of the mixture after 24 h of incubation [15]. All assays were executed in triplicate, and the gathered data underwent statistical verification using ANOVA paired with Duncan’s multiple range test as previously described.

### 2.8. Determination of Probiotic Properties

#### 2.8.1. Hemolytic Activity of *B. megaterium*

To evaluate the hemolytic potential of *B. megaterium*, isolates were inoculated onto blood agar plates supplemented with 5% (*v*/*v*) horse blood (Thermo Scientific, Oxoid Microbiology Products) and maintained at 37 °C for a 24-h incubation period. Positive hemolytic activity was identified by the formation of a distinct translucent zone surrounding the bacterial colonies [16].

#### 2.8.2. Determination of Bile Salt and Acid Tolerance

To evaluate the resilience of *B. megaterium* against bile salts across a concentration spectrum of 0% to 10%, an assay was conducted using modified nutrient broth (NB) formulations with various concentrations of bile salts [17]. Specifically, 100 µL aliquots of the bacterial suspension (optimized to a density of 10^7^–10^8^ CFU/mL) were inoculated onto MRS agar supplemented with corresponding levels of bile salts (ranging from 0% to 10%). Following a 24-h incubation period at 37 °C, the plates were inspected for the development of bacterial lawns to establish the maximum tolerance threshold for each tested concentration.

For acid tolerance, nutrient broth was prepared at different pH conditions (1.5–6.5) with 5 M HCl. Then 1 mL of overnight *B. megaterium* culture (OD_600_ of approximately 1.0) was added to 19 mL of prepared NB. The samples were then incubated at 37 °C for 24 h. The growth was measured by spectrophotometry at 600 nm. A *t*-test was performed at a 95% confidence interval to determine the statistical significance (*p* < 0.05).

#### 2.8.3. Antibiotic Susceptibility Analysis

To evaluate antibiotic susceptibility, a broth microdilution methodology was implemented [18]. The panel of tested antimicrobial agents, comprised of ampicillin, ciprofloxacin, kanamycin, streptomycin, erythromycin, vancomycin, tetracycline, and chloramphenicol, was obtained from Sigma-Aldrich (St. Louis, MO, USA). Working stock solutions were generated at concentrations of 4 and 40 mg/mL to prepare twofold serial dilutions across 96-well microtiter plates. Each well was pre-filled with 100 µL of sterile nutrient broth (NB) to facilitate the dilution series. Subsequently, *B. megaterium* suspensions were introduced into the wells to achieve an initial inoculum density of 10^7^ to 10^8^ CFU/mL. Following a 24-h incubation at 37 °C, a microplate reader was used to measure optical density at 600 nm [19].

## 3. Results and Discussion

Initially, *B. megaterium* was isolated from a coffee fermentation field in Thailand. The crude supernatant collected from the culture was then evaluated for antimicrobial activity against the tested foodborne pathogens, including *B. cereus*, *E. coli*, and *S. enterica* subsp. *enterica* Typhimurium, using the disc diffusion method. This revealed the supernatant to have a relatively broad spectrum of activity, though different degrees of inhibition against each tested foodborne pathogen. Specifically, a greater antimicrobial effect was observed against the growth of *B. cereus* and *E. coli* than *S. enterica* subsp. *enterica* serovar Typhimurium. Against *B. cereus* and *S. enterica* subsp. *enterica* serovar Typhimurium, the supernatant achieved approximately half the inhibitory effect of kanamycin and erythromycin; however, against *E. coli*, its effect was approximately the same as erythromycin (Table 1). Biochemical analysis of the crude extract identified it to contain diverse bioactive compounds, including ethylenediaminetetraacetic acid, betaine, N-butanoyl-L-homoserine lactone, phenylethylamine, δ-valerolactam, ε-caprolactam, maculosin, xamoterol, norharman, and triacanthine, which collectively represent antioxidant, anti-inflammatory, quorum sensing, antimicrobial activity, and therapeutic activities [20,21,22,23,24,25,26,27].

δ-Valerolactam and ε-caprolactam, in particular, have been reported to possess bactericidal and antimicrobial activities [28,29]. Therefore, the antimicrobial activity from the crude extract of *B. megaterium* could be caused by the action of δ-valerolactam and ε-caprolactam (Table 2). However, *B. megaterium* has been reported to produce several other key antimicrobial metabolites, the most notable and primary of which are megacins, lipopeptides, and oxetanocin. Because of its diverse metabolic pathways, *B. megaterium* serves as a powerful biological control agent against both plant and animal pathogens [30].

Valerolactam and caprolactam have never been reported to be produced from *B. megaterium*. δ-valerolactam is a derivative of the β-lactam antibiotic that has been reported to react under the same reactivities as anti-infections to various pathogens [7]. A derivative of valerolactam has been shown to inhibit the adhesion of *E. coli* in bladder epithelial cells [32]. Both valerolactam and caprolactam can be used as monomers for the synthesis of industrial polyamides, including high-value nylon-5 and nylon-6,5, and can function as antibacterial polymers to kill *Staphylococcus aureus* (Gram-positive) and *Escherichia coli* (Gram-negative) [33,34]. ε-Caprolactam was also found to affect ecologically important soil bacteria, for instance, inhibiting the growth of several *Bacillus* species [15]. However, valerolactam and caprolactam have antimicrobial properties; they have not been typical components reported in standard probiotic strains.

**Figure 1:**
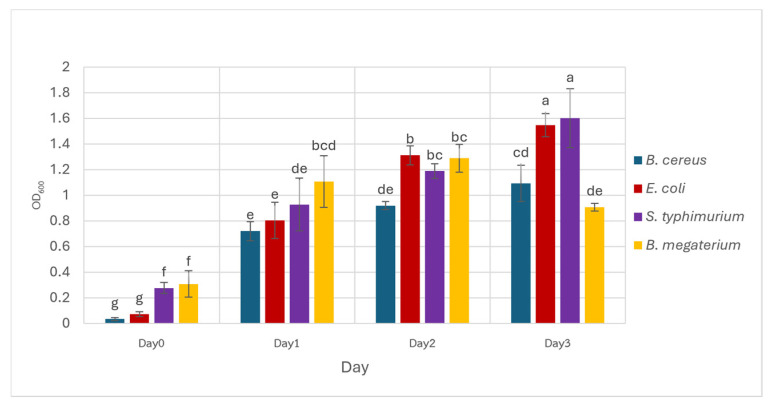
Indirect measurement of surface-attached biomass by crystal violet staining (A600) for cultures of *B. megaterium*, *B. cereus*, *E. coli*, and *S. enterica* subsp. *enterica* serovar Typhimurium in PS tubes for 3 days. Error bars indicate the standard deviation (*n* = 3). Different letters represent significant difference at *p* < 0.05, determined by ANOVA.

**Figure 2:**
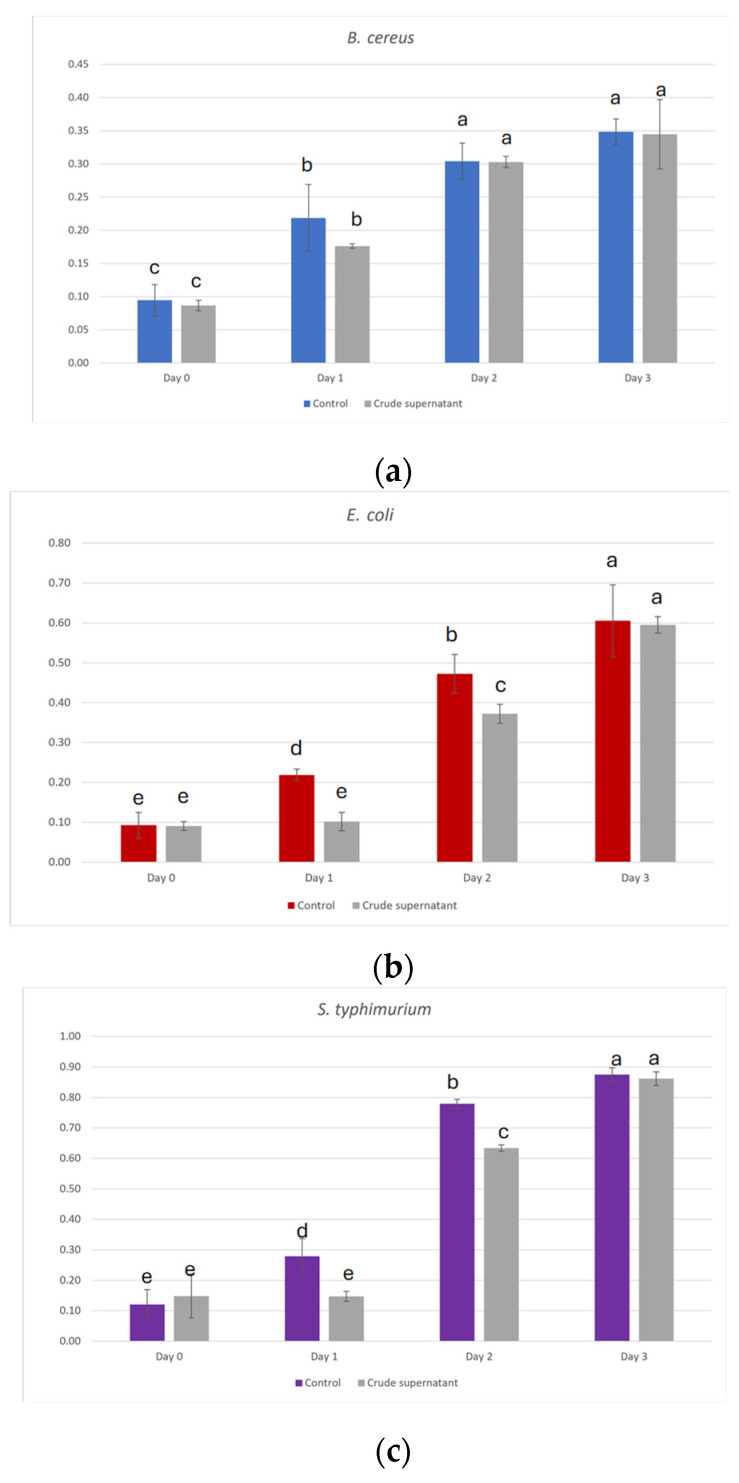
Antimicrobial activity of *B. megaterium* crude supernatant against biofilm formation of *B. cereus* (**a**), *E. coli* (**b**), and *S. enterica* subsp. *enterica* serovar Typhimurium (**c**) over three days, determined by crystal violet staining. Error bars indicate the standard deviation (*n* = 3). Different letters indicate significant difference at *p* < 0.05, determined by ANOVA.

Biofilm development by probiotic strains reportedly exerts a barrier effect, thereby suppressing foodborne pathogenic biofilm formation and mitigating disease transmission [15,35]. Therefore, a key research focus is developing probiotic technologies that leverage biofilm-mediated resistance against foodborne pathogens and production of antimicrobial compounds. In this work, biofilm formation by *B. megaterium*, *B. cereus*, *E. coli*, and *S. enterica* serovar *enterica* Typhimurium was evaluated over a three-day period using a crystal violet staining assay to quantify bacterial attachment or biofilm formation on PS tubes as surfaces mimicking the intestinal mucosa surface, which simultaneously replicates the hydrogel barrier properties of intestinal mucus and supports healthy mammalian cell attachment (Figure 1). Specifically, staining intensity correlated with the extent of bacterial attachment and subsequent biofilm formation on PS tubes [36,37], allowing spectrophotometric quantification of attachment. During the three-day incubation period, bacterial attachment increased steadily for all strains except *B. megaterium*, exhibited a decline on day 3 that was likely to be caused by the biofilm detachment. The detection of N-butanoyl-L-homoserine lactone within the crude culture supernatant highlights its role as a key autoinducer, confirming its capacity to drive biofilm development [38]. Furthermore, the *B. megaterium* crude supernatant was tested against foodborne pathogenic biofilm development over three days. Significant biofilm inhibition occurred against *B. cereus* on day 1, and against *E. coli* and *S. enterica* subsp. *enterica* serovar Typhimurium on day 2. For all test foodborne pathogens, this inhibitory effect was restricted to the initial phase of the bacterial attachments (Figure 2). The inhibition of pathogen biofilm formation could be mediated by the presence of antimicrobial metabolites in the crude supernatant, including δ-valerolactam and ε-caprolactam (Table 2). Derived lactams have been investigated for their effect against various species of oral biofilms and pathogenic biofilm due to their ability to inhibit bacterial biofilm formation and modulate quorum sensing [39,40]. Crude supernatant derived from *B. megaterium*, containing N-butanoyl-L-homoserine lactone, may significantly influence bacterial attachment and subsequent biofilm formation in our tested foodborne pathogens. *Bacillus* sp. QSI-1 has been documented to modulate quorum sensing signaling pathways, effectively reducing the colonization of fish gut pathogens like *Aeromonas hydrophila* by prompting their detachment from the host intestinal mucosa. However, the exact molecular mechanisms underlying this signal disruption and subsequent bacterial detachment remain fully elucidated [41].

**Figure 3:**
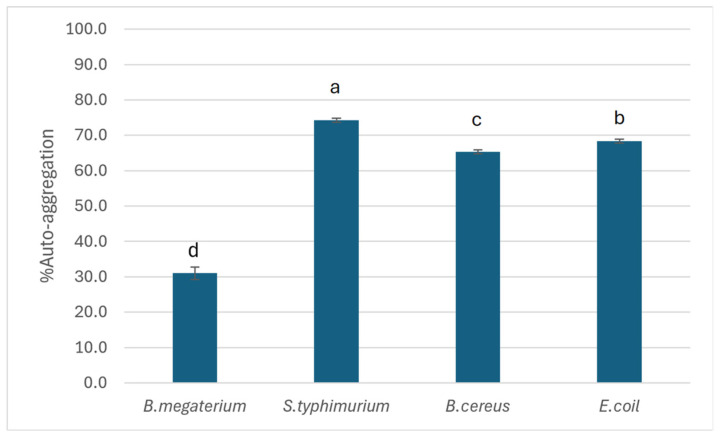
Percent auto-aggregation of *B. megaterium* and pathogenic bacteria after 24 h of incubation at 37 °C. Data represents the mean of three replicates. Error bars represent standard deviation. Different letters represent significant difference at *p* < 0.05, determined by ANOVA.

**Figure 4:**
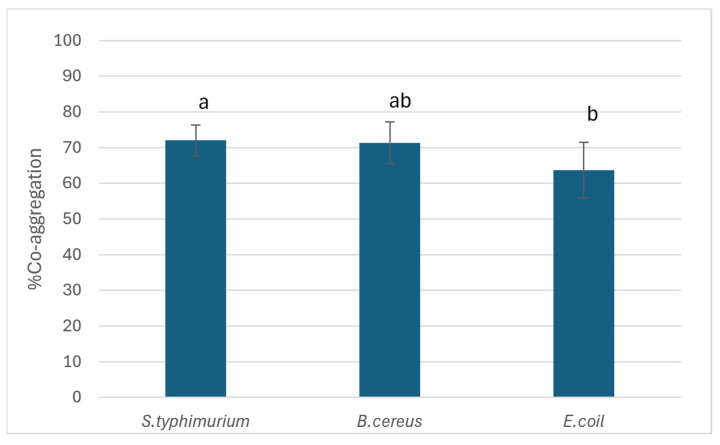
Percent co-aggregation of *B. megaterium* with pathogenic bacteria after 24 h of incubation at 37 °C. Data represents the mean of three replicates. Error bars represent standard deviation. Different letters represent significant difference at *p* < 0.05, determined by ANOVA.

Aggregation rate is another parameter that can indicate the potential to prevent intestinal tract colonization by foodborne pathogenic bacteria [42]. The aggregation assays in this study determined the percent auto-aggregation of pathogenic *B. cereus, E. coli*, and *S. enterica* subsp. *enterica* serovar Typhimurium to be 65%, 68%, and 74% respectively; meanwhile, the value for *B. megaterium* was only 31% (Figure 3). Co-aggregation assays of *B. megaterium* with the tested pathogens obtained co-aggregation values of 72% for *S. enterica* subsp. *enterica* serovar Typhimurium, 71% for *B. cereus* and 63% for *E. coli*, and (Figure 4). This could be interpreted as the presence of *B. megaterium* slightly hindering the colonization of the tested foodborne pathogens, which could imply an important role for *B. megaterium* in preventing foodborne pathogen from colonizing and infecting host cells, especially in the gastrointestinal tract [42]. Collectively, these results suggest that *B. megaterium* could prevent adhesion of foodborne pathogenic bacteria through co-aggregation or cell adhesion blockage as well as by secreting active compounds (as represented by the crude supernatant) that effectively inhibit biofilm formation by the foodborne pathogens.

**Figure 5:**
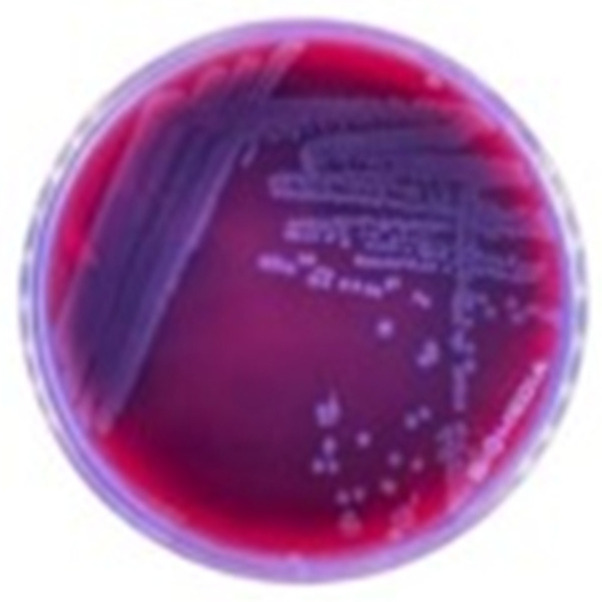
Hemolytic activity assay showing no activity by *B. megaterium*.

**Figure 6:**
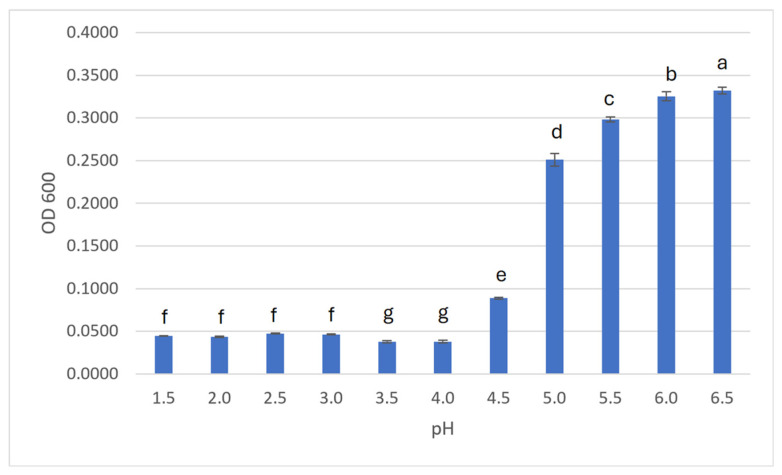
Growth of *B. megaterium* on medium with pH 1.5–6.5 incubated at 37 °C for 24 h. Data represents the mean of three replicates. Error bars represent standard deviation. Different letters represent significant difference at *p* < 0.05, determined by ANOVA.

To assess the potential of *B. megaterium* as a probiotic, its physiological and microbial characteristics were determined, including hemolytic activity, acidic pH tolerance, bile salt tolerance, and antibiotic susceptibility (Figure 5 and Figure 6). *B. megaterium* was able to tolerate bile up to a concentration of 6% and did not show hemolytic activity (Table 3, Figure 5). Other probiotic strains reportedly tolerate 4% bile salts [43]. With regard to pH, *B. megaterium* showed optimal growth at pH 6.5, with a significant drop at pH 4.5 and lower. However, some bacterial still survived at pH values as low as 1.5. This is a critical criterion, as a viable probiotic strain must withstand the highly acidic environment of the human stomach. Given that effective probiotic strains must withstand a minimum pH of 3.0, the capacity of *B. megaterium* to tolerate both acidic conditions and bile salts suggests it can survive passage through the human gastrointestinal tract.

For the antibiotic susceptibility assay, we determined the minimum inhibitory concentrations (MICs) of ampicillin, ciprofloxacin, kanamycin, streptomycin, erythromycin, vancomycin, tetracycline, and chloramphenicol (Table 4). The MIC values obtained for chloramphenicol, vancomycin, streptomycin, gentamicin, ampicillin, and kanamycin did not exceed the values suggested by the European Food Safety Authority (EFSA) for *Bacillus* spp. to be used as a probiotic [44].

Taken together, *B. megaterium* exhibited promising probiotic characteristics and would not pose a risk to consumers, as demonstrated by its negative hemolytic activity and susceptibility to standard antibiotics. The strain effectively inhibited the planktonic growth, co-aggregation, and biofilm development of the tested foodborne pathogens. Furthermore, it produces a variety of broad-spectrum antibacterial compounds capable of targeting both Gram-positive and Gram-negative pathogens in both planktonic and biofilm forms. While these findings support the potential application of *B. megaterium* as a probiotic, comprehensive future studies are required to fully characterize its probiotic properties. Specifically, further investigation should include screen-testing for known *Enterocin* genes, whole-genome analysis, evaluating its adherence to human intestinal cells, assessing cholesterol assimilation and lipolytic activity, and validating its survival within a simulated gastrointestinal tract [45,46,47].

## 4. Conclusions

*B. megaterium* strain isolated from a coffee fermentation field was analyzed for its potential development as a probiotic. The strain demonstrated the capacity to produce diverse bioactive compounds that effectively inhibited the planktonic growth and biofilm development of multiple foodborne pathogens. Furthermore, the strain demonstrated strong competitive exclusion capabilities. When analyzed on a polystyrene surface mimicking the intestinal mucosa, both direct physical blockage by the biofilm and the secretion of its active compounds successfully prevented the attachment of foodborne pathogens.

The strain also exhibited desirable probiotic traits, including tolerance to bile salts and acidity, a non-hemolytic profile, and susceptibility to standard antibiotics. Additionally, it produced diverse bioactive compounds with promising therapeutic potential. Therefore, *B. megaterium* represents a promising candidate for development as a probiotic supplement for humans and animals, although further characterization of its probiotic properties, including in vivo testing as probiotics for human and animal feed, genetic testing, whole genome sequencing, and PCR-based assessment of antibiotic-resistance genes, remains necessary before conferring usage. Notably, this work is the first to demonstrate both the antimicrobial activity and antibiofilm-forming ability through the production of bioactive compounds, including δ-valerolactam and ε-caprolactam by *B. megaterium*, highlighting its potential utility in food preservation, sanitization within the food processing industry, and medical therapeutics. Therefore, future purification and identification of the active compound will enable its practical application.

## Figures and Tables

**Table 1:** Antimicrobial activity of the crude supernatant from *B. megaterium* culture against *B. cereus*, *E. coli*, and *S. enterica* subsp. *enterica* serovar Typhimurium as determined using the disc diffusion method measuring the inhibition zone (mm). Different lowercase superscripts denote significant differences (*p* < 0.05).

	Water	Kanamycin	Erythromycin	*B. megaterium* Crude Supernatant
*B. cereus*	No inhibition	14.33 ± 1.13 ^a^	12.58 ± 0.76 ^b^	6.92 ± 0.52 ^d^
*E. coli*	No inhibition	11.42 ± 0.38 ^b^	6.92 ± 1.18 ^d^	7.17 ± 1.23 ^d^
*S. typhimurium*	No inhibition	11.67 ± 0.29 ^b^	8.67 ± 0.29 ^c^	3.08 ± 1.53 ^e^

**Table 2:** Biochemical compounds detected in the crude supernatant from *B. megaterium* using UHPLC and mass spectrometry.

RT	Name	Function	References
0.52	(Z)-3-((1H-benzo[d]imidazol-2-yl)imino)isoindolin-1-one	-	-
0.52	Ethylenediaminetetraacetic acid	Medical benefit in the treatment of psoriasis as an antioxidant	[20]
0.68	Betaine	Anti-inflammatory functions in numerous diseases	[21]
0.76	4-(dimethylamino)azobenzene n-oxide	-	-
0.94	N,N-diethyl-3-(2-methyl-5-pyridin-4-yl-1,2,4-triazol-3-yl)azetidine-1-carboxamide	-	-
0.95	Hypoxanthine	-	-
0.96	9-[[5-(2-methoxyphenyl)-1H-pyrazol-4-yl]methyl]-1-oxa-9-azaspiro [5.5]undecan-4-ol	-	-
1.07	(3S,6S)-3-Methyl-6-(2-methylpropyl)piperazine-2,5-dione	-	-
1.14	L-Alloisoleucine	-	-
1.28	2-(3,4-dimethoxyphenyl)-1-[2-[3-(3,4-dimethoxyphenyl)-1,2,4-oxadiazol-5-yl]pyrrolidin-1-yl]ethenone	-	-
1.3	2-Pyrrolidinone	-	-
2.98	2-[5-[(4-fluorophenoxy)methyl]-1,2,4-oxadiazol-3-yl]-N-propan-2-ylpyrrolidine-1-carboxamide	-	-
3.01	N-butanoyl-L-homoserine lactone	Signal molecule in quorum sensing system	[31]
3.37	2-(4-benzylpiperazin-1-yl)-N-(4-phenyl-1,3-thiazol-2-yl)acetamide	-	-
3.39	Phenylethylamine	Potential to treat mental disorders	[22,26]
3.45	δ-Valerolactam	Bactericidal activity	[27]
5.14	Ethyl 2-[4-[[2-(3,5-dimethyl-1,2-oxazol-4-yl)pyrrolidine-1-carbonyl]amino]phenyl]acetate	-	-
5.4	ε-Caprolactam	Antibacterial activity	[29]
5.75	N-(2-methylphenyl)-2-(4-methylphenyl)imidazo [1,2-a]pyridin-3-amine	-	-
6.47	Maculosin	Antioxidant	[23]
6.49	1-(1-acetyl-2,3-dihydroindol-5-yl)-4-(1,4-dioxa-8-azaspiro [4.5]decane-8-carbonyl)pyrrolidin-2-one	-	-
6.5	2-(phenoxymethyl)-1-[[1-(pyridin-2-ylmethyl)piperidin-4-yl]methyl]benzimidazole	-	-
7.7	7-Ethyl-N-[(5-methylfuran-2-yl)methyl]-2-pyridin-2-ylpyrazolo [1,5-a]pyrimidine-5-carboxamide	-	-
7.7	Xamoterol	Treatment of heart failure	[24]
8.22	(3S,6S)-3-methyl-6-(2-methylpropyl)piperazine-2,5-dione	-	-
8.74	1-(4-methylphenyl)-5-oxo-N-propan-2-ylpyrrolidine-3-carboxamide	-	-
9.11	1-Propanoyl-2,3-dihydroindole-5-carboxamide	-	-
9.42	3-(tert-butylamino)-N-[(3-fluorophenyl)methyl]spiro [1H-pyrido [2,3-b]pyrazine-2,3’-pyrrolidine]-1’-carboxamide	-	-
9.48	1-Cyclopentyl-3-[2-[5-(piperidine-1-carbonyl)benzotriazol-1-yl]ethyl]urea	-	-
9.85	Norharman	Potential neuroprotective agent	[25]
9.95	1-Ethyl-3-[2-[4-(3-fluoro-4-methylphenyl)sulfonylpiperazin-1-yl]ethyl]pyrrolo [2,3-b]pyridine	-	-
9.95	2-[4-[1-(4-methylphenyl)pyrazol-4-yl]-3,6-dihydro-2H-pyridin-1-yl]-1-(4-pyridin-2-ylpiperazin-1-yl)ethenone	-	-
9.95	L,L-cyclo(leucylprolyl)	-	-
10.68	4-Formyl Indole	-	-
10.83	Indole-2-acetic acid	-	-
11.47	(3aS,6aR)-5-(1H-indol-5-ylmethyl)-2-methyl-3a,4,6,6a-tetrahydropyrrolo [3,4-c]pyrrole-1,3-dione	-	-
12.27	N-ethyl-1-(3-methylphenyl)-5-oxopyrrolidine-3-carboxamide	-	-
12.38	Triacanthine	Antitumor effect	[26]
12.6	N-ethyl-N-methylcathinone	-	-

**Table 3:** Determination of *B. megaterium* bile salt tolerance the concentration of 0–10%.

Concentration	0%	2%	4%	6%	8%	10%
Microbial growth (+/−)	+	+	+	+	−	−

**Table 4:** Minimum inhibitory concentration (µg/mL) of various antibiotics against *B. megaterium*; experiment performed in triplicate.

AMP	CIP	KAN	STR	ERY	VAN	TET	CMP
<1.25	<1.25	<1.25	<1.25	<1.25	<1.25	<1.25	2.5

AMP, ampicillin; CIP, ciprofloxacin; KAN, kanamycin; STR, streptomycin; ERY, erythromycin; VAN, vanomycin; TET, tetracycline; and CMP, chloramphenicol.

## Data Availability

The raw data supporting the conclusions of this article will be made available by the authors on request.
